# Low expression of PRDM5 predicts poor prognosis of esophageal squamous cell carcinoma

**DOI:** 10.1186/s12885-022-09787-8

**Published:** 2022-07-07

**Authors:** Jing Guo, Qiuxing Yang, Sheng Wei, Jingjing Shao, Tianye Zhao, Liyuan Guo, Jia Liu, Jia Chen, Gaoren Wang

**Affiliations:** 1 Affiliated Tumor Hospital of Nantong University, Nantong Tumor Hospital, Nantong, Jiangsu China; 2 Cancer Research Center Nantong, Affiliated Tumor Hospital of Nantong University, Nantong Tumor Hospital, Nantong, Jiangsu China; 3 Department of Oncology, Affiliated Tumor Hospital of Nantong University, Nantong Tumor Hospital, Nantong, Jiangsu China; 4 Department of Radiation Oncology, Affiliated Tumor Hospital of Nantong University, Nantong Tumor Hospital, Nantong, Jiangsu China

**Keywords:** PRDM5, Esophageal squamous cell carcinoma, Prognosis, WNT signaling pathway, Immunohistochemical, DNA methylation

## Abstract

**Background:**

The role of the PRDM5 in esophageal squamous cell carcinoma (ESCC) has not been revealed. This study investigated the relationship between PRDM5 expression and survival outcome in esophageal squamous cell carcinoma and explored the mechanism in tumor development.

**Methods:**

In present study, expression of PRDM5 mRNA in esophageal squamous cell carcinoma patients was conducted using the Cancer Genome Atlas (TCGA) and Gene Expression Omnibus (GEO) database. The expression of PRDM5 was assessed by immunohistochemical staining. Kaplan-Meier curve and Cox regression analysis was performed to analyze the survival outcome and independent predictive factors. qRT-PCR and Methylation-specific PCR were performed to identify the mRNA level of PRDM5 and Methylation rate. Cibersort algorithm to analyze the relationship between PRDM5 expression and immune cell invasion. Western-blot was performed to confirm the expression of esophageal tumor tissues and adjacent tissues.

**Results:**

The TCGA database and GEO database show that PRDM5 mRNA level in esophageal squamous cell carcinoma adjacent tissues was higher than that of cancer tissues, and ESCC patients with high expression of PRDM5 mRNA had better overall survival. Tissue microarray showed that the protein level of PRDM5 in the adjacent tissues of patients with ESCC was higher than that in cancer tissues, and the expression level of PRDM5 was significantly correlated with the grade of clinicopathological characteristics (*P* < 0.001). Patients with high expression of PRDM5 displayed a better OS and DFS. Cox regression analysis showed that PRDM5 was an independent risk factor and prognostic factor for ESCC patients (HR: 2.626, 95%CI: 1.824–3.781; *P* < 0.001). The protein level of PRDM5 matched with the transcriptional level, whereas the DNA methylation affected the transcriptional level. Cibersort showed that T cells CD4 memory resting, mast cells resting, eosinophils, M2 macrophages and mast cells activated were significantly positively correlated with PRDM5 expression (*P* < 0.05), while regulatory T cells, monocytes and dendritic cells negatively correlated with PRDM5 expression (*P* < 0.05).

**Conclusion:**

PRDM5 can be used as a biomarker to predict the survival of ESCC patients. Furthermore, PRDM5 expression in ESCC cells may affect WNT/β-catenin signaling pathways, thus further affect the ESCC cell proliferation, migration, and invasion capacity.

**Supplementary Information:**

The online version contains supplementary material available at 10.1186/s12885-022-09787-8.

## Background

Esophageal cancer is one of the most common cancers in the world, which seriously threatens human life and health. According to the latest global cancer statistics, the incidence and mortality of esophageal cancer ranked ninth and sixth respectively [1]. In China, the incidence and mortality of esophageal cancer rank third and fourth among all malignant tumors respectively [2]. Therefore, esophageal cancer has always been the main malignant tumor threatening the health of Chinese residents. Due to the obvious regional differences in the incidence of esophageal cancer, the incidence of ESCC is higher than esophageal adenocarcinoma. Although we have made great progress in the diagnosis and treatment of esophageal cancer in recent years, due to the high recurrence rate after treatment and the limitations of drugs and treatment strategies after metastasis. Therefore, the overall survival rate of ESCC is still disappointing in china. There is still a lack of reliability markers to guide and predict the prognosis of esophageal cancer. Most patients with esophageal cancer are ESCC in china. Therefore, there is an urgent need for effective and independent markers to predict clinical prognosis. This study aimed to investigate whether PRDM5 might be a biomarker for the prognosis of ESCC and explore the mechanism in tumor development.

PRDM5 belongs to PRDM family which is a PR range zinc finger domain and quantity structure (except PRDM11) [3]. The PRDM protein N at the end of the PR domain is 20 to 30% similar to SET and belongs to the SET protein subtype [4]. SET has histone lysine protein transferase activity, which can change the chromosome structure and affect the form of epigenetic reprogramming, therefore may affect gene expression [5]. According to the mode of action, Zinc finger protein can bind to DNA specifically, regulate the transcription factor, and have transcriptional activity. Zinc finger protein can also mediate the interaction between proteins and participate in the post-transcriptional regulation process such as the ripening, clipping and degradation of mRNA, showing RNA binding properties [6, 7]. The PRDM family of proteins has an unequal number of C2H2 zinc finger protein structures, while PRDM5 has 1 PR domain and 16 zinc finger structures [8].

PRDM5 has a PR/SET domain at the N-terminal, but non-histone methyltransferase (HMTase) activity. In contrast, PRDM5 acts as an epigenetic modifier by recruiting histone-modifying enzymes such as HMTase G9A and HDAC1 to the target gene [9]. It was a stress response gene that exists as a protective factor when it is not silenced by methylated [10]. Silencing PRDM5 by methylation in tumor cell lines leads to changes in various tumors including nasopharyngeal, esophageal, gastric, hepatocyte, and cervical cancer. Whereas PRDM5 methylation was seldom detected in lung, colon, ovarian, and bladder cancer cell lines [10]. Immunostaining showed nuclear expression of infected cells indicating that PRDM5 is a nuclear protein [11]. As a transcription factor, CHIP analysis found that PRDM5 directly binds to CDK4 and TWIST1 promoters. In addition, PRDM5 expresses the CDK4 and TWIST1 promoters of H3K4me3 and the levels of acetyl histone H4 are significantly reduced [10]. CHIP sequencing of colorectal cancer cell lines found that the response genes Adamts9, Col1a1, Mmp13 and Mgll in PRDM5 have been confirmed strongly regulated by PRDM5 [12]. Further exploration of the downstream pathways can reveal that PRDM5 can be regulated by WNT/β-catenin signal, and as an epigenetic modifier of the expression of a variety of cancer genes to exert its tumor suppressor effect.

Furthermore, PRDM5 also served as an early diagnostic and prognostic marker, while detection of PRDM5 DNA methylation in gastric secretion may be used as a diagnose method in early gastric cancer [13]. PRDM5 methylation is also an early event in the occurrence and development of BRAF mutant colorectal cancer. In addition, PRDM5 protein levels were significantly reduced in both mutant and wild-type intestinal polyps, especially in BRAF mutant and wild-type cancers. This indicates that PRDM5 down-regulation may start in the early stages of tumor development and continue with the development of the disease [14]. In another research, PRDM5 expression levels in glioma specimens were low and correlated with clinicopathological parameters and poor prognosis [15]. However, the protein level of PRDM5 in ESCC and the correlation between survival has not been studied so far. All the above evidence suggests that PRDM5 plays a key role in the carcinogenesis and development of tumors, and guides us to further explore whether PRDM5 acts as a tumor suppressor gene in ESCC and the prognostic significance of PRDM5.

## Methods

### Bioinformation analysis of public datasets TCGA & GEO

PRDM5 mRNA levels and corresponding information of samples were downloaded from TCGA (https://www.cancer.gov/) and GEO database (http://www.ncbi.nlm.nih.gov/GEO/). The database was collected under the following conditions: (1) containing at least 10 cases of normal tissue; (2) PRDM5 expression data were extracted comprehensively. In addition, two ESCC data sets were downloaded from database: GSE53624 and GSE53622. Both the TCGA and GEO databases contain information on the clinical characteristics and prognosis information of patients with ESCC.

### Human esophageal squamous cell carcinoma specimens and clinical data collection

Tissue microarrays were obtained from 2 groups of independent samples of patients with esophageal squamous cell carcinoma from 2 medical centers. The discovery sets included 169 patients underwent curative esophagectomy obtained from the First People’s Hospital of Nantong City from 2010 to 2014. Due to the poor quality of tissue microarrays and the lost of patient’s clinical information, and 154 cases were used for research. Among them, there were 147 paired tissues between cancer and adjacent cancer. The validation group included 279 patients underwent curative esophagectomy from 2013 to 2014 in the Affiliated Hospital of Nantong University. Due to the poor quality of tissue microarrays and the lost of patient’s clinical information, there were 255 cases for research. Clinical baseline data were collected for each patient retrospectively. None of the patients received any of the preoperative anticancer treatment such as preoperative chemotherapy, radiotherapy and immunotherapy before surgery, and concurrent radical esophageal squamous cell carcinoma surgery. All the information of patients including age, sex, tumor size, depth of invasion (T), lymph node status (N), metastasis (M), and overall survival (OS) were recorded from computerized medical records. Using of human tissues with informed consent was confirmed by each patient. This research was approved by the Clinical Research Ethics Committee of each hospital.

### Immunohistochemistry (IHC) staining and scoring

Formalin-fixed paraffin-embedded surgical specimens were performed for construction of tissue microarray (TMA). Tissue blocks were sectioned at 2 mm and prepared on glass slides. A subsequent immunohistochemistry study was conducted to identify expression of PRDM5. The immunohistochemical protocols were performed as below. TMA was firstly fixed by formaldehyde and repaired by EDTA, then hydrogen peroxide was used to extinguish endogenous HRP. After rinsing, the primary PRDM5 antibody (purchased from abcam) was diluted (1:1000) with antibody diluent. The primary antibody was taken out the next day and washed three times and then incubated with a secondary antibody (rabbit 1:2000 Proteintech) for 20 minutes.

All specimens were independently reassessed by 2 pathologists according to the International Union against Cancer TNM classification system. The intensity grades of staining are described as follows: negative (0), weak (1), medium (2), and strong (3), while the degree of staining was scored according to the percentage of positive cells: 0(0%); 1(1–25%); 2(26–50%); 3(51–75%); and 4(76–100%). The combined score was calculated by multiplying the intensity and density of staining eventually. Low expression of PRDM5 is defined as a score of 1–6, and high expression of PRDM5 is defined as a score of ≥7.

### Quantitative real-time PCR (qRT-PCR)

A total of 15 pairs of esophageal tumor and para-tumor tissues were collected from surgical specimens of patients underwent radical surgery of esophageal tumor in Affiliated Tumor Hospital of Nantong University. Total RNA was extracted using Trizol (Sigma) according to the manufacturer’s instructions, and then 1μg of obtaining cDNA was extracted for reverse transcription and detect the expression level of endogenous PRDM5 mRNA. Accurate quantification was achieved using the standard curve, which was produced by continuously diluting a known amount of RNA from the in vitro transcription response and using the dilution series for TaqManqPCR with the patient sample. Quantitative analysis of mRNA expression was conducted using the StepOnePlus™ Real-Time PCR System. The primers and TaqMan probes for analysis were designed using the manufacturer’s software, PrimerExpress, and the PRDM5 primers were supplied by and. The reference gene GAPDH is used as an internal control for RNA quality. All quantitative analyses were repeated to assess the consistency of the results. Relatively standardization of the target relative to the GAPDH gene expression level calculation for Δ Ct = Ct (target) - Ct (GAPDH). The relative quantification values of PRDM5 were calculated using the 2 ^-△△CT^ method.

### Methylation-specific polymerase chain reaction (MSP)

By methylation-specific polymerase chain reaction (methylation-specific polymerase chain reaction, MSP) method, collection of esophageal squamous cell carcinoma patients who resection of carcinoma tissue and paired normal tissue adjacent to carcinoma, using tissue genomic DNA extraction kit (QIAGEN GmbH) to extract DNA, the DNA methylation kit (ZYMO RESEARCH) for DNA denaturation and bisulfite conversion. The transformed DNA was used as a template for PCR amplification. PRDM5 methylation-specific primers and non-methylation-specific primers (Servicebio) were designed. The methylation-specific primers were upstream 5-TTGTTTCGGGTTTCGCGTTC-3 and downstream 5′-ATTCCTACTACGAAAACGCG-3′. PRDM5 geneno-methylation-specific primers were upstream 5′-TAGTTTTGTTTTGGGTTTTGT-3′ and downstream 5′-CCATTCCTACTACAAAAA CACA-3′. PCR reaction system: DNA template 1 L, 5 × PCR buffer 10 L, upstream and downstream primers 0.5 L each, with ribozyme free water added up to 20 L. PCR reaction conditions: pre-denaturation at 95 °C for 120 s, denaturation at 95 °C for 60s, annealing at 55 °C for 60s, elongation at 72 °C for 60s, 40 cycles. PCR products were analyzed by the gelatinization imaging system. For example, the target band amplified by gene methylation-specific primers indicated positive methylation of the PRDM5 gene, and the gene methylation rate was calculated.

### Western blot analysis

A total of 15 pairs of esophageal tumor and para-tumor tissues were collected from surgical specimens of patients underwent radical surgery of esophageal tumor in Affiliated Tumor Hospital of Nantong University. Extract tissue protein: Take out fresh esophageal cancer and adjacent tissues from the refrigerator at − 80 °C, rewarm on ice, and then weigh 0.1 g of each sample into a 1.5 mL EP tube, add 1 mL Mix the protein lysate. The lysate was mixed by RIPA Lysis Buffer, PMSF, 5x loading according to 1:100:4 and ice bath for 10 minutes. Then put the lysate in a biological sample homogenizer, fully homogenize and ice bath for 30 minutes, transfer to a low-temperature centrifuge, set at 4 °C, 12000 rpm, and centrifuge for 20 minutes. After centrifugation, transfer the supernatant to a new EP tube. Prepare the protein loading solution according to 5× loading buffer: protein lysate = 1:4, pipette and mix well, dry at 100 °C Boil in a thermostat for 10 minutes, and centrifuge again at 4 °C and 12,000 rpm for 10 minutes. Finally, determine the protein concentration in each sample and record it. Store it at − 20 °C. When used, take it out and boil for 3 minutes. Centrifuge at 4 °C and 12,000 rpm for 3 minutes before loading. Sample Proteins were separated by 10% sodium dodecyl sulfate-polyacrylamide gel electrophoresis and transferred to a polyvinylidene fluoride membrane (microporous). At room temperature, the membrane was enclosed in 5% bovine serum albumin for 1 hour. The membrane was incubated with a primary antibody at 4 °C overnight, GAPDH (1:500, #66009–1-1 g; Proteintech), PRDM5 (1:1000, # ab7609; Abcam) were washed three times with TBST and incubated at room temperature with horseradish peroxidase-conjugated secondary antibodies for 1 hour. TBST was used three times and ECL (bio-rad) visualized.

### Immune cell infiltration analysis

A deconvolution algorithm based on CIBERSORT gene expression (http://cibersort.stanford.edu/) [16]was used to evaluate the immune cell infiltration in 179 patients obtained from the GEO database (http://www.ncbi.nlm.nih.gov/GEO/). The samples were divided into two groups according to the average expression of PRDM5, Wilcoxon Signed Rank test was used to compare immune cell content between groups with high and low expression of PRDM5 mRNA. Heatmaps and violin plots were analyzed and plotted using the packages “pheatmap” and “vioplot”, respectively.

### Statistical analysis

All statistical analyses were performed using the SPSS19.0 software (SPSS, Chicago, Illinois) and GraphPad Prism 5 software (GraphPad Software, Inc.) R software (http://www.r-project.org/) was used to extract and standardize mRNA expression data from these databases, and then GraphPad Prism Version 8.0 (GraphPad Software, Inc.) was used for statistical analysis. The correlation between PRDM5 gene expression and copy number variation and DNA methylation was analyzed using online database cBioPortal (http://www.cbioportal.org/) and MEXPRESS (http://mexpress.be/). The paired or unpaired Student’s t-test was performed to analyze the statistical significance between two groups. Cox regression model was used to determine the risk factors of OS through univariate and multivariate analysis. The association between expression levels and progression-free survival in Esophageal squamous cell carcinoma patients were analyzed by Kaplan–Meier survival curves, using Kaplan-Meier Plotter. Log-rank test was used for statistical analysis. A *P*-value< 0.05 was considered statistically significant.

## Results

### Esophageal tumor tissues exhibit decreased PRDM5 mRNA expression

**Fig. 1:**
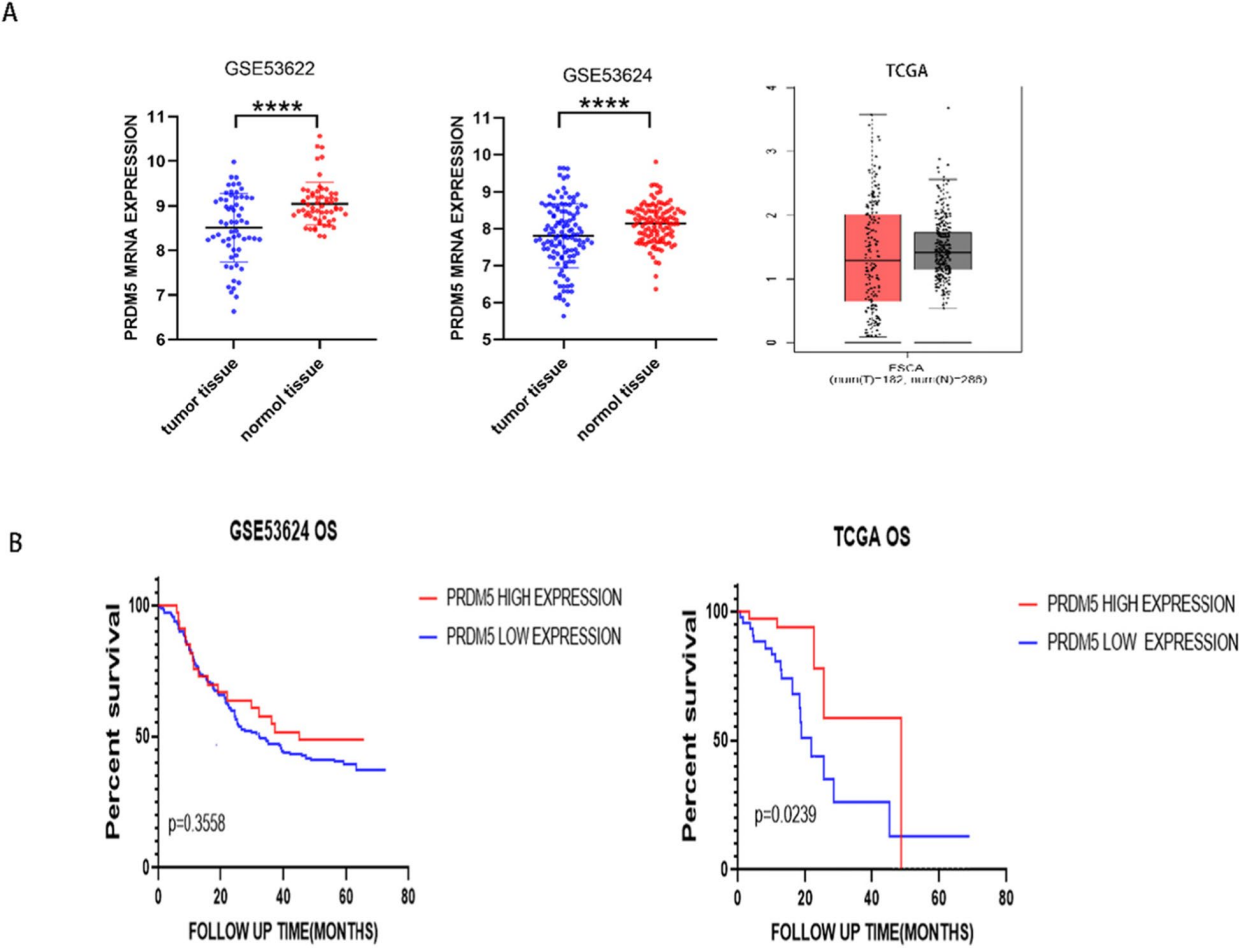
PRDM5 mRNA level in esophageal cancer. **A** PRDM5 mRNA in GSE53622, GSE53624, TCGA database. **B** PRDM5 mRNA level and OS in esophageal cancer

PRDM5 mRNA levels and corresponding information of samples were downloaded from TCGA and GEO database. From TCGA database and GSE53622, GSE53624 two data sets, it was found that PRDM5 mRNA level in esophageal tumor tissue was significantly lower than para-tumor tissue (Fig. 1A).

### Correlation of PRDM5 mRNA levels with overall survival (OS) in esophageal tumor patients

Based on the TCGA database, patients of Esophageal squamous cell carcinoma with high PRDM5 mRNA levels displayed longer OS than those with low expression (*P* = 0.0254) (Fig. 1b). Whereas, although there is no statistical significance between the high mRNA levels of PRDM5 expression with the OS in GEO data sets, we did observe the trend of a prolonged OS from the point of view of survival analysis (Fig. 1B).

### Patients’ characteristics and immunohistochemical in TMA

**Fig. 2:**
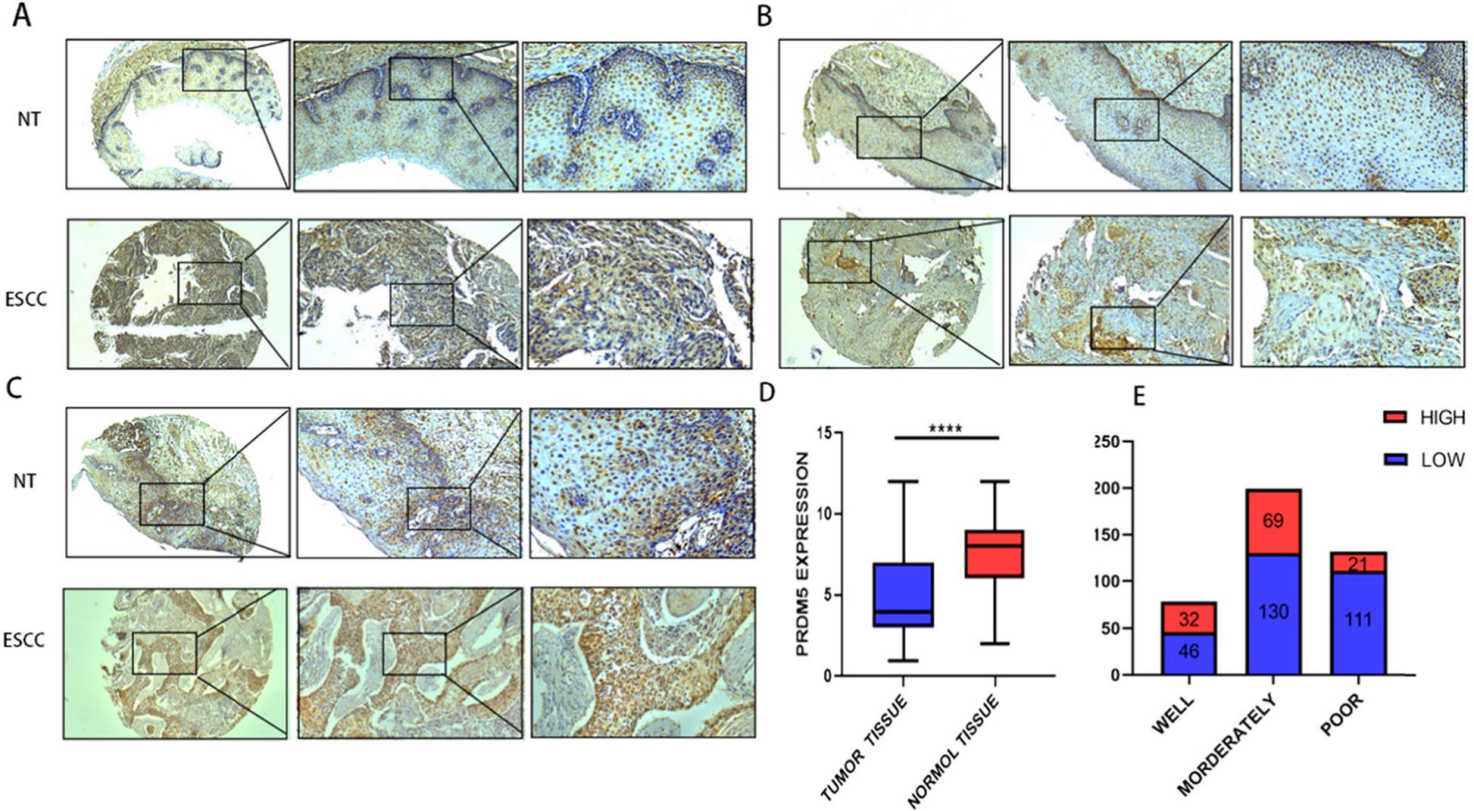
Representative pictures of PRDM5 expression in ESCC tissues (left50×, middle100×, right20×). **A** Poorly differentiated carcinoma and para-cancer tissue. **B** Moderately differentiated carcinoma and para-cancer tissue. **C** Highly differentiated carcinoma and para-cancer tissue. **D** Protein level of PRDM5 carcinoma and para-cancer tissue. **E** The expression of PRDM5 in esophageal carcinoma with well, middle and poor differentiation

Patient characteristics and pathological and clinical features are shown in Table 1. To investigate the relationship between PRDM5 and prognosis of Esophageal squamous cell carcinoma, immunohistochemistry was performed to evaluate the expression level of PRDM5 in tissue microarray (TMA) of 409 patients (discovery data set 154, validation data set 255). Esophageal squamous cell carcinoma tissues and adjacent tissues were paired in the discovery set. As indicated before, the combined score was calculated by multiplying the intensity and density of staining eventually. Low expression of PRDM5 was defined as a score of 0–6, while high expression of PRDM5 was defined as a score of ≥7. It was found that the expression of PRDM5 in adjacent tissues was higher than in tumor tissues (Fig. 2D). Furthermore, it was also shown that PRDM5 expression was lower in poorly differentiated esophageal squamous cell carcinoma and on the contrary in highly differentiated esophageal squamous cell carcinoma (Fig. 2A-C). The immunohistochemical staining showed a high expression of PRDM5 displayed 29.2% in the discovery data set and 30.1% in the validation data set (Fig. 2E). Detailed characteristics are summarized in Tables 1 and 2.

**Table 1:** Relationship between PRDM5 expression and clinicopathological characteristics in Discovery Data Set

	PRDM5 LOW	PRDM5 HIGH	*P*-value
Patient characteristics	*N* = 109	*N* = 45	
Sex			0.872
female	28	11	
male	81	34	
Age			0.864
≧60	79	32	
< 60	30	13	
Tumorinformation Location			0.866
Upper	7	2	
Middle	50	20	
Lower	52	23	
Tobacco			0.812
N0	89	36	
YES	20	9	
Alcohol			0.331
N0	93	41	
YES	16	4	
T stage			0.105
I-II	38	22	
III-IV	71	23	
N stage			0.091
N0	59	31	
YES	50	14	
M stage			0.360
N0	107	45	
YES	2	0	
TNM stage			0.122
I-II	63	32	
III-IV	46	13	
Histological grade			< 0.001
Well	3	7	
Moderately	65	35	
Poorly	41	3	
Accompanying diseases			0.933
N0	89	37	
YES	20	8	
Positive margin			
N0	101	41	0.744
YES	8	4	
Nerve invasion			0.178
N0	87	40	
YES	22	5	
Vascular invasion			0.013
N0	83	42	
YES	26	3

**Table 2:** Relationship between PRDM5 expression and clinicopathological characteristics in Validation Data Set

	PRDM5 LOW	PRDM5 HIGH	*P*-value
Patient characteristics	*N* = 178	*N* = 77	
Sex			0.177
female	54	17	
male	124	60	
Age			0.496
≧60	134	61	
< 60	44	16	
T stage			0.012
I-II	67	35	
III-IV	111	42	
N stage			0.061
N0	93	50	
YES	85	27	
M stage			0.540
N0	177	76	
YES	1	1	
TNM stage			0.013
I-II	95	54	
III-IV	83	23	
Histological grade			0.030
Well	19	14	
Moderately	89	45	
Poorly	70	18	
Nerve invasion			0.232
N0	167	75	
YES	11	2	
Vascular invasion			0.045
N0	147	71	
YES	31	6	

### The correlation of PRDM5 exprssion and DFS, OS

**Fig. 3:**
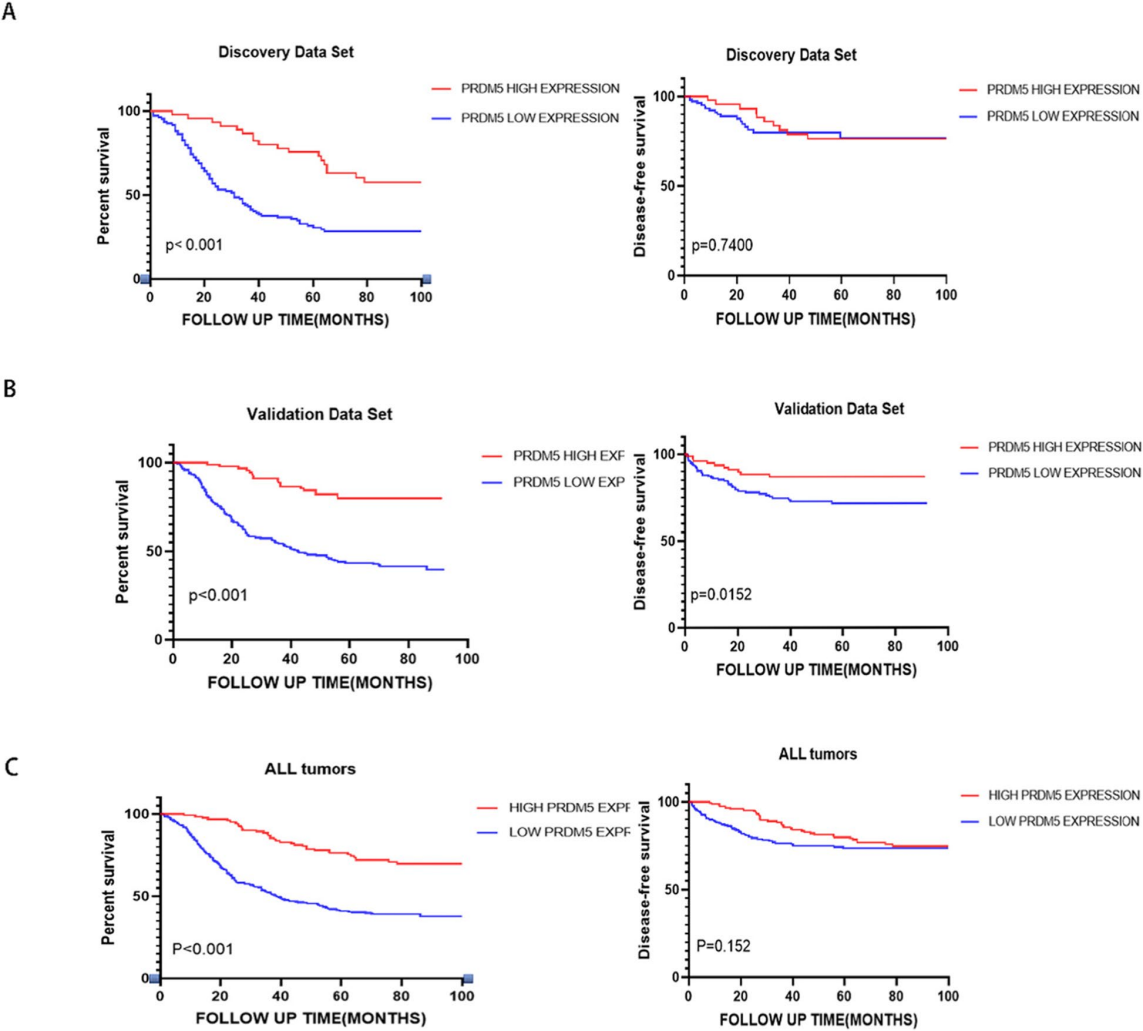
Relationship between PRDM5 expression and OS/DFS in esophageal cancer patients. **A** Discovery Data Set. **B** Validation Data Set. **C** Combined Data Set

In the discovery data set, we analyzed the relationship between the expression of PRDM5 and the DFS and OS of patients. As indicated in Fig. 3A, patients with high expression of PRDM5 had prolonged DFS and OS comparing with patients with low expression. It was either shown in the validation set and the combined data set (Fig. 3B, C). Besides, the expression levels of PRDM5 were related to TNM staging, tumor differentiation, and vascular invasion in the combined data set (Table 3). Through univariate analysis, we found that T stage (HR: 0.573, 95%CI: 0.43–0.764; *P* < 0.001), N stage (HR: 0.526, 95%CI: 0.401–0.69; *P* < 0.001), TNM staging (HR: 0.509, 95%CI: 0.388–0.667; *P* < 0.001), PRDM5 expression level (HR: 2.986, 95%CI: 2.084–4.278; *P* < 0.001), differentiation degree (HR: 0.724, 95%CI: 0.537–0.976; *P* = 0.034), nerve infiltration (HR: 0.643, 95%CI: 0.429–0.964; *P* = 0.0.033), and vascular invasion (HR: 0.497, 95%CI: 0.359–0.688; *P* < 0.001) were risk factors of ESCC patients. Multivariate analysis revealed the independent prognostic factors for ESCC patients were expression of PRDM5 (HR: 2.626, 95%CI: 1.824–3.781; *P* < 0.001), T stage (HR: 0.719, 95%CI: 0.533–0.969; *P* = 0.0.03), TNM stages (HR: 0.657, 95%CI: 0.49–0.881; *P* < 0.001), and vascular invasion (HR: 0.694, 95%CI: 0.493–0.976; *P* = 0.0.036) (Table 4).

**Table 3:** Relationship between PRDM5 expression and clinicopathological characteristics in Combined Data Set

	PRDM5 LOW	PRDM5 HIGH	*P*-value
Patient characteristics	*N* = 287	*N* = 122	
Sex			0.241
female	82	28	
male	205	94	
Age			0.668
≧60	74	93	
< 60	213	29	
T stage			0.003
I-II	105	57	
III-IV	182	65	
N stage			0.012
N0	152	81	
YES	135	41	
M stage			0.832
N0	284	121	
YES	3	1	
TNM stage			0.004
I-II	158	86	
III-IV	129	36	
Histological grade			< 0.001
Well	22	21	
Moderately	154	80	
Poorly	111	21	
Nerve invasion			0.073
N0	254	115	
YES	33	7	
Vascular invasion			0.002
N0	230	113	
YES	57	9

**Table 4:** Univariate and multivariate analysis of characteristics associated with overall survival

Variables	Univariate analysis				Multivariate analysis			
	Hazard Ratio	95%CI		***P***-value	Hazard Ratio	95%CI		***P***-value
Age	1.041	0.764	1.418	0.798				
Sex	0.889	0.653	1.212	0.458				
T stage	0.573	0.43	0.764	< 0.001	0.719	0.533	0.969	0.03
N stage	0.526	0.401	0.69	< 0.001				
M stage	0.385	0.143	1.037	0.059				
TNM stage	0.509	0.388	0.667	< 0.001	0.657	0.49	0.881	< 0.001
Histological grade	0.724	0.537	0.976	0.034				
PRDM5	2.986	2.084	4.278	< 0.001	2.626	1.824	3.781	< 0.001
Vascular invasion	0.497	0.359	0.688	< 0.001	0.694	0.493	0.976	0.036
Nerve invasion	0.643	0.429	0.964	0.033				

### Stratified analysis of OS in esophageal tumor patients

**Fig. 4:**
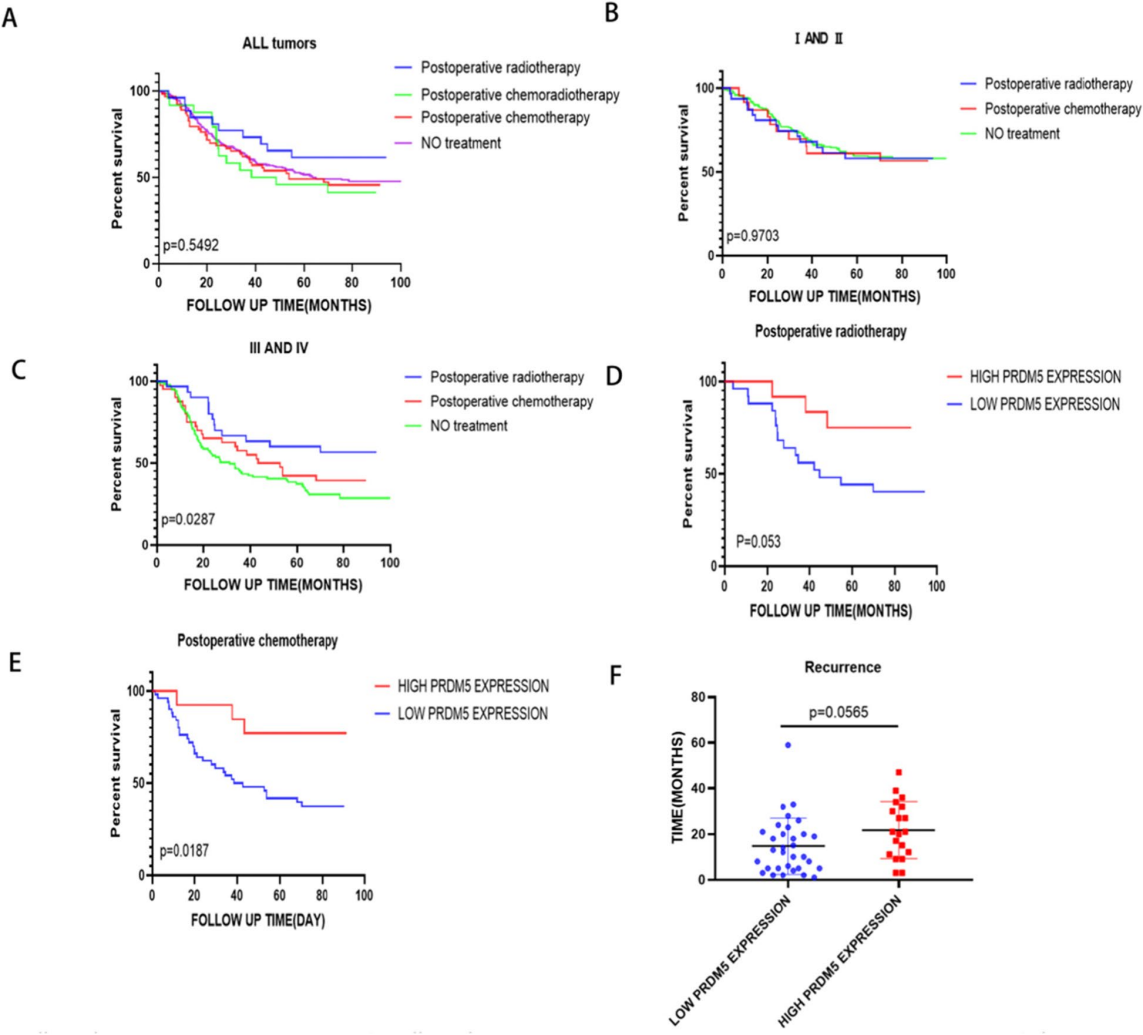
Effects of PRDMA5 and postoperative treatment on overall survival (OS). **A** postoperative treatment and OS. **B** Postoperative treatment and OS in stage I-II. **C** Postoperative treatment and OS in stage III-IV. **D** PRDM5 expression and OS in patients underwent postoperative radiotherapy. **E** PRDM5 expression and OS in patients underwent postoperative chemotherapy. **F** PRDM5 expression and RFS

Detailed research was conducted to investigate the correlation of several factors related to overall survival in esophageal tumor patients. Patients were stratified into several groups according to factors as below: postoperative radiotherapy, postoperative chemoradiotherapy, postoperative chemotherapy, no treatment. As shown in Fig. 4A, postoperative radiotherapy could prolong the OS in total group, while the postoperative chemoradiotherapy didn’t affect the OS. In Fig. 4B, postoperative therapy didn’t affect the OS in stage I-II Esophageal squamous cell carcinoma patients. Whereas, in stage III-IV Esophageal squamous cell carcinoma patients, postoperative treatment could extend the OS especially in patients who underwent postoperative radiotherapy (Fig. 4B, C). Moreover, the high expression level of PRDM5 was associate with longer OS in patients who underwent postoperative therapy (Fig. 4D, E). The analysis based on the relapse-free survival (RFS) showed there are significant differences among the low expression PRDM5 and the high group (Fig. 4F).

### Prognostic nomogram and calibration plots of esophageal squamous cell carcinoma

Based on the results of univariate and multivariate analysis, we constructed a nomogram to predict the operating system at 3-year, 5-year, and 10-year after surgery (Supplementary Fig. 1). According to preliminary analysis, predictive factors include T status, vascular invasion, tumor grade, T stage, TNM staging, and PRDM5 staining, all of which were important prognostic indicators of OS.

### The transcriptional level of PRDM5 was consistent with the protein level and was affected by methylation

**Fig. 5:**
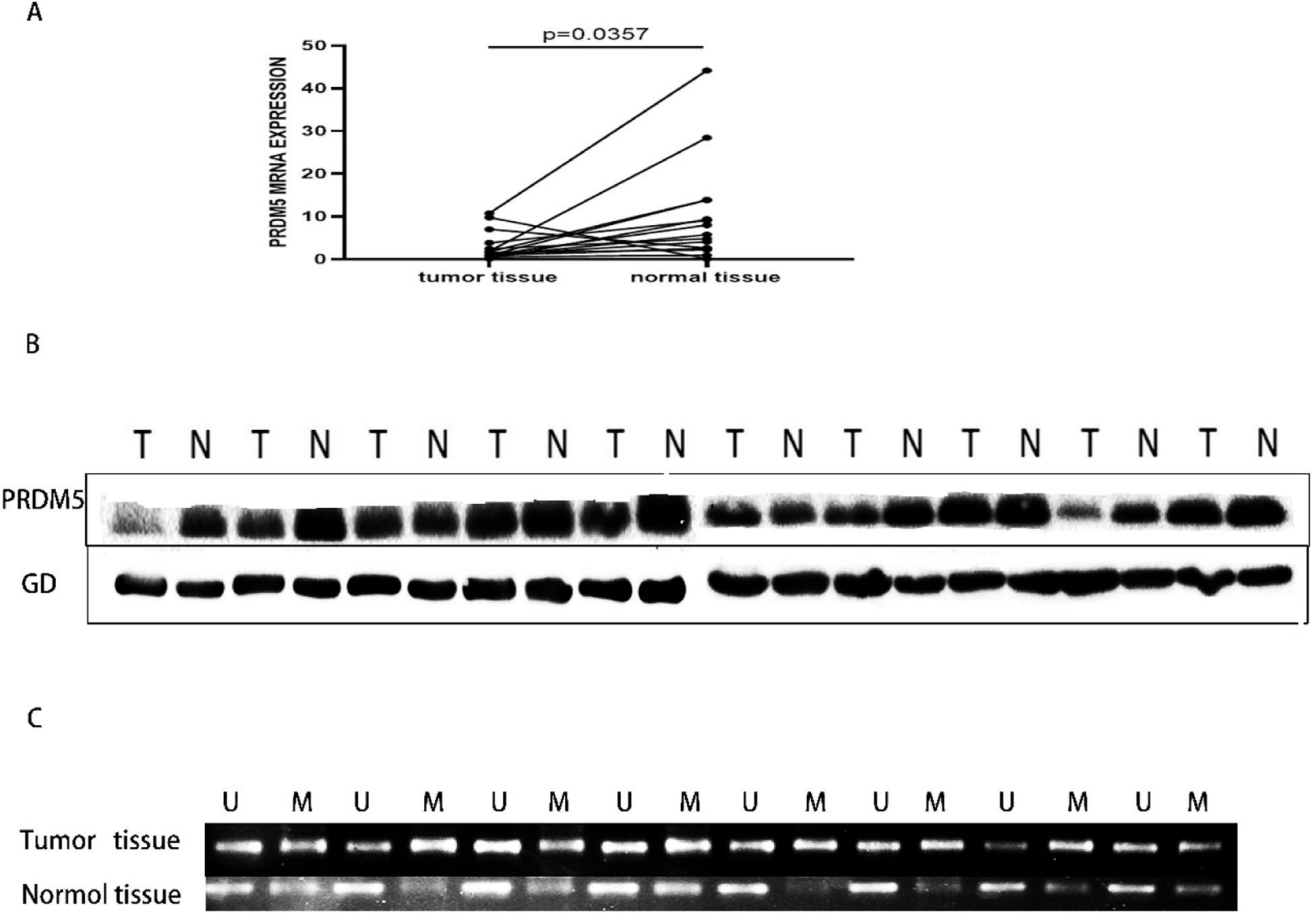
PRDM5 expression in adjacent tissues and tumor tissues. **A** mRNA level in adjacent tissues and tumor tissues. **B** Protein level of PRDM5 in adjacent tissues and tumor tissues. **C** Methylation-specific PCR in adjacent tissues and tumor tissues

**Fig. 6:**
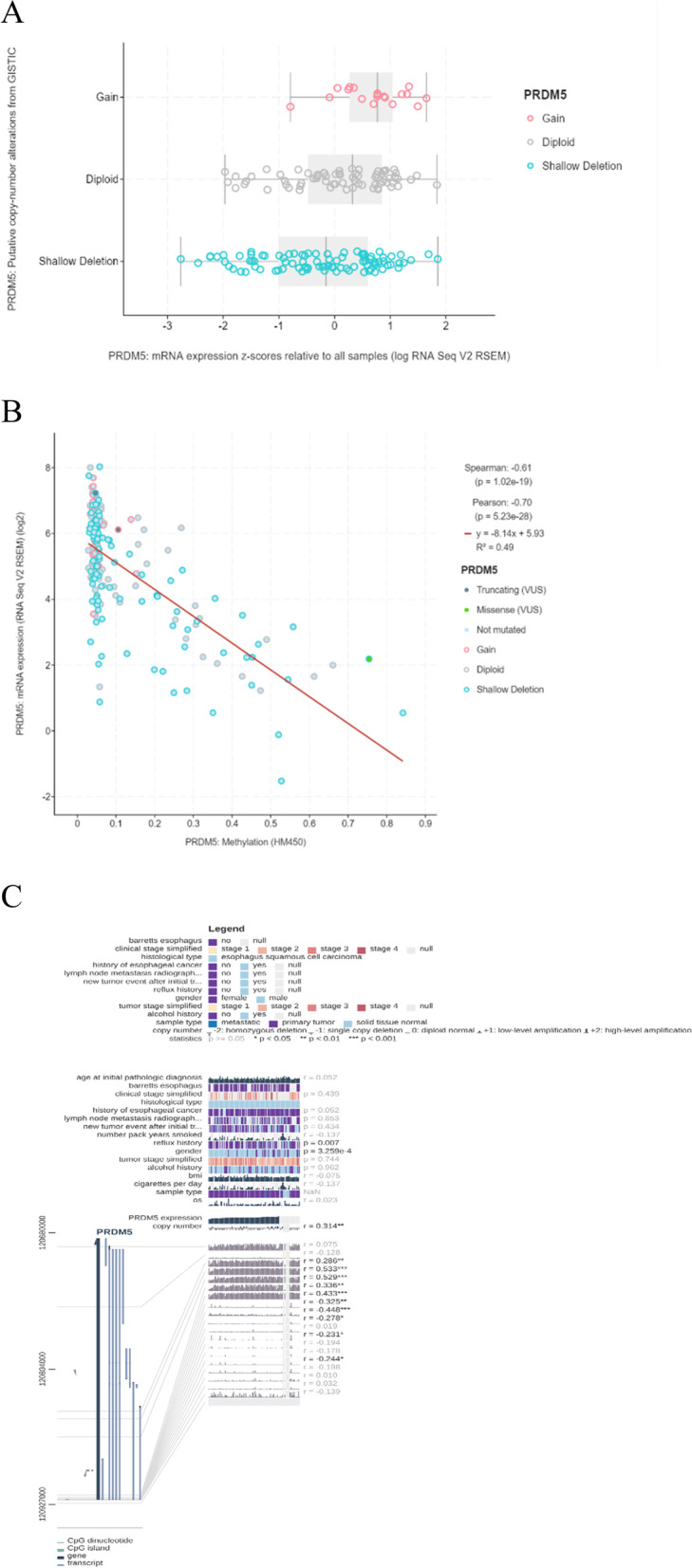
The relationship between PRDM5 in online databases and methylation. **A** The relationship between PRDM5 methylation and DNA copy number. **B** The relationship between PRDM5 methylation and mRNA. **C** PRDM5 methylation site prediction

Next, the expression of PRDM5 of esophageal tumor tissues and paired para-tumor tissues were analyzed. It was found that the expression of PRDM5 was lower in 15 specimens of esophageal tumor than para-tumor tissues (Fig. 5A). We selected 10 pairs of tissues with high expression of PRDM5-mRNA level in para-tumor tissues, Western-blot was performed using 10 pairs of cancer and adjacent tissues to detect protein expression levels and found the protein level of PRDM5 in adjacent tissues was higher than that tumor tissues, which proved that PRDM5 had consistency in both the transcription level and protein level changes (Fig. 5B). We then performed methylation PCR on eight pairs of Esophageal squamous cell carcinoma tissue and found that PRDM5 DNA was highly methylated in cancer tissues, and absent in adjacent tissues (Fig. 5C). Therefore, we further investigated the relationship between PRDM5 DNA methylation and copy number in ESCC in cBioPortal. There was no significant difference between PPRDM5 DNA methylation and copy number (Fig. 6A). However, there was a significant negative correlation between PRDM5-mRNA expression and DNA methylation, which was confirmed by Pearson correlation coefficient (Spearman: -0.61, *P* = 1.02^− 19^) (Fig. 6B). We also found in the MEXPRESS (http://mexpress.be/) database that methylation at CPG position 120,921,881 was negatively correlated with PRDM5-mRNA expression (Fig. 6C). Therefore, all these data suggested that methylation at CPG Island 120,921,881 (the promoter of PRDM5) leads to down-regulation of PRDM5 mRNA expression, which in turn leads to a decrease in PRDM5 protein level.

### PRDM5 is associated with immune infiltration of ESCC

**Fig. 7:**
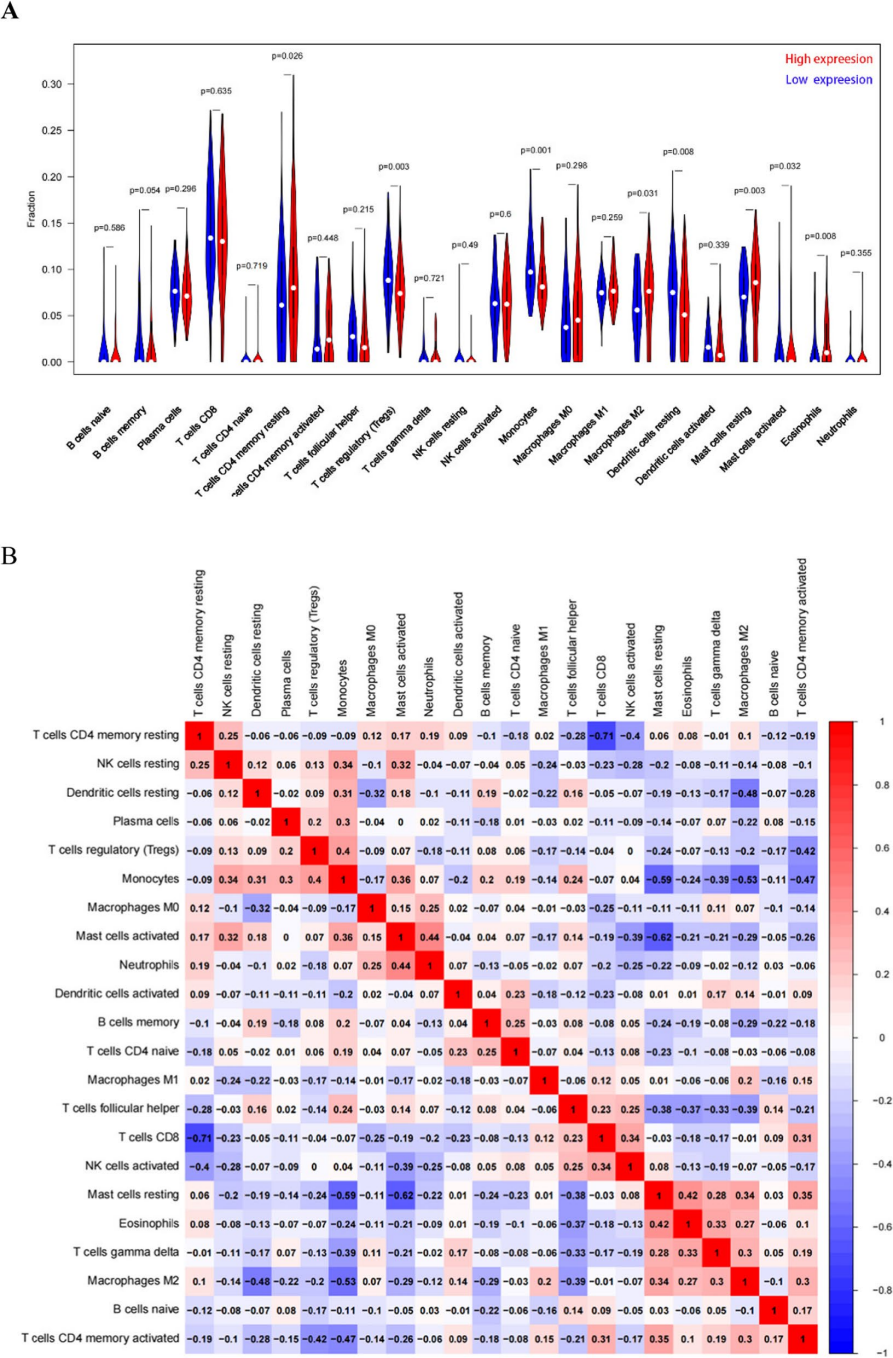
The relationship between the expression of PRDM5 and immune infiltration in patients with esophageal squamous cell carcinoma. **A** The ratio of 22 subtypes of immune cells in the high and low PRDM5 expression groups in esophageal squamous cell carcinoma samples is different. **B** Heat map of 22 immune infiltrating cells in esophageal squamous cell carcinoma samples

We also used the Cibersort algorithm to analyze the expression levels of 22 immune cell subgroups and evaluate their correlation with PRDM5 expression. The results showed that T cells CD4 memory resting, Mast cells resting, Eosinophils, M2 macrophages and Mast cells activated were significantly positively correlated with PRDM5expression (*P* < 0.05). T cells regulatory (Tregs), Monocytes, and Dendritic cells resting were negatively correlated with PRDM5 expression (Fig. 7A). In addition, we evaluated possible associations between 22 immune cells, and heat maps showed weak to moderate correlations in the rates of different tumor-infiltrating immune cell subgroups (Fig. 7B).

## Discussion

In this study, we did research on the clinical role of PRDM5. Based on this research, we found that the expression of PRDM5 in esophageal para-tumor tissue was significantly higher than in tumor tissues, regardless of mRNA level or protein level, and esophageal squamous cell carcinoma patients with high expression of PRDM5 had a better prognosis. Recent studies indicated that high levels of PRDM5 are associated with better outcomes for glioma and colorectal cancer [14, 15]. Our research showed that postoperative chemotherapy is necessary for esophageal patients with stage III-IV after surgery, and postoperative radiotherapy significantly prolonged the patient’s OS. However, postoperative adjuvant treatment for patients with stage I-II Esophageal squamous cell carcinoma after surgery has little benefit on OS, the results also suggested that postoperative adjuvant treatment for patients with high expression of PRDM5 can help to prolong DFS and OS. This might indicate that PRDM5 could affect the therapeutic effect.

Previous studies have shown that ectopic expression of PRDM5 can inhibit the proliferation of gastric cancer, nasopharyngeal cancer, and brain glioma cells [10, 15]. Our study showed that the expression of PRDM5 was correlated with pathological status T stage. It also demonstrated that over-expression of PRDM5 inhibited proliferation activity in esophageal carcinoma cell. This aroused our thinking, indicating that the patient’s pathological information such as the high expression of PRDM5 should be considered comprehensively when choosing postoperative adjuvant treatment and patients treatment strategies. However, since it is difficult to obtain dynamic monitoring only by immunohistochemical methods, we suggest that it might be more helpful to detect PRDM5 expression in patients’ serum.

This experiment proved that PRDM5 transcriptional level and protein level were consistent, and the transcriptional level was affected by methylation. Previous studies have found that PRDM5 expression is lower in multiple cancer species than in adjacent tissues, including breast, ovarian, liver, lung, colon cancers, and cervical cancers [10], and that silencing or downregulation of PRDM5 is often caused by DNA methylation [10, 12, 14, 17]. And then extended to epigenetics, studies have shown that long-term heavy drinking can leading to homocysteine and S-adenosine homocysteine (SAH) increment, resulting in histone modifications and changes in gene expression. Eventually affect the small RNA family which serve as epigenetic regulators causing DNA promoter methylation [18]. In this study, esophageal squamous cell carcinoma patients with drinking history were relatively small proportion, so the history of drinking was not statistically significant. However, we could find that the PRDM5 expression was often lower in patients with a long-term history of drinking, which may be related to DNA methylation. Studies have shown that PRDM5 is a stress response gene [10]. It may provide protection when the esophagus is physically or chemically stimulated, but once methylation is silenced or its expression is reduced, it may lead to the occurrence and development of esophageal squamous cell carcinoma. We also found in database that methylation at CPG position 120,921,881 was negatively correlated with PRDM5 mRNA expression, suggesting that methylation is negatively correlated with PRDM5 mRNA expression. In future studies, we would confirm whether this site causes the silencing or reduction of PRDM5 expression. It is worth considering whether blocking the methylation at certain site should be considered in clinical applications for patients with low PRDM5 expression. The methylation of PRDM5 may be involved in the early events of esophageal squamous cell carcinoma, such as esophagitis. Then this may also be used as a biomarker for the early diagnosis of esophageal squamous cell carcinoma. Future research should expand the sample size of esophageal squamous cell carcinoma and add patient samples from patients with esophagitis for further research. We believe that in the future, PRDM5 methylation may also become part of cancer screening, and there is a blood-based screening for DNA methylation markers in colorectal cancer [19], we showed that epigenetic therapy of PRDM5 is achievable. In the future, we anticipate targeted therapies will also include epigenetically inactivated tumor inhibitors, such as the CRISPRDCAS9 technology and the viral application of the epigenetic editor to reactivate. Our results showed that T cells CD4 memory resting and eosinophilia activated were significantly positively correlated with PRDM5expression. Monocytes and Dendritic cells resting were negatively correlated with PRDM5 expression. Recent studies have shown that CD4+ T cells are not a pure cell lineage with a single function, but a diverse cell population with complex functions. In addition, CD4+ T cells can be used not only as helper cells, but also as effective effector cells or partners with macrophages and eosinophils to eliminate various tumors, and Patients with increased CD4+ T cell ratio in serum samples of patients with esophageal squamous cell carcinoma undergoing radiotherapy have better survival [20]. High systemic CD4 memory T cell counts assessed by peripheral blood samples prior to initiation of PD-L1/PD-1 blockade therapy can serve as a reliable predictive biomarker for NSCLC patients, in addition, dynamic changes in CD4 T cell memory populations can be successfully used. In “real-time” monitoring of blood sample responses during immunotherapy to identify early progressors and patients at high risk of progression, the enhancement of systemic CD8 antitumor responses by PD-L1/PD-1 inhibitors may be coordinated by peripheral CD4 T cells [21, 22]. In the study of melanoma, eosinophilia was a favorable prognostic factor for OS, probably due to the prevention of loss of tumor antigen expression and T cell exhaustion, eosinophilia can be induced by acting as antigen-presenting cells. It secretes chemoattractants to activate new T cells and recruit them to the tumor site to prevent acquired drug resistance [23]. Monocytes significantly increase the level of glycolysis in the peritumoral region of human HCC. Activation of glycolysis induces PD-L1 expression on these cells and subsequently attenuates cytotoxic T lymphocyte responses in tumor tissue, dendritic cells induce peripheral CD8+ T cell tolerance through PD-1 and CTLA-4 [24], and in cervical cancer. In the study, the tumor-infiltrating CD8+ T cells of patients with high dendritic cells showed a trend of inactivation, insufficient cytotoxicity, and a depleted phenotype [25, 26]. Whether esophageal cancer patients with high PRDM5 expression can enhance the efficacy of PD-1 inhibitors by affecting the corresponding immune cells will be worthy of our further study.

PRDM5 also plays an essential role in many aspects such as cell cycle and cell function. Besides, PRDM5 also regulates the G2/M phase cell cycle [9, 11, 15]. Silencing PRDM5 alone can reduce the apoptosis of glioma cells and intestinal epithelial cells [15, 27]. Ectopic PRDM5 expression can significantly reduce the colony formation efficiency of all cancer cells in monolayer culture [10, 15]. PRDM5 overexpression significantly inhibits the activity of the TOP flash reporter molecule, which further inhibits WNT/β-catenin signal conversion, thereby inhibiting the activity of its downstream target gene CCND1 [4, 10, 28], which in turn affects the changes of CDK4 [10]. Studies have shown that CDK4 can phosphorylate Smad3, which is very important in response to TGF-β signal transcription activity. Phosphorylation of Smad3 by CDK4 inhibits its anti-proliferation function [29]. CDK4 also directly phosphorylates FOXM1 at multiple sites to positively regulate its activity, possibly protecting cancer cells from senescence by inhibiting reactive oxygen species (ROS) levels, and promoting the entry of G1/S phase by regulating the expression of several genes in cancer cells (such as cyclin E2, MYB and MCM2) [30]. CDK4 can phosphorylate MEP50 and increase methyltransferase activity and cell survival function [31]. Our previous results confirmed that the expression level of PRDM5 is related to the pathological characteristics of tumor size, then we speculate whether the expression of PRDM5 affected CDK4 and thus affected the proliferation ability of esophageal squamous cell carcinoma cells. We further speculate that PRDM5 may be involved in the CCND1-CDK4/CDK6 pathway, and that patients with low expression of PRDM5 may increased the expression levels of CCND1 and CDK4. It is worth exploring whether CDK4/CDK6 inhibitors (Palbociclib) can be considered for this type of patients. We will further verify its relationship with CCND1 and CDK4 in future cell function experiments, which is of great significance for clinical treatments.

The previous results indicate that the expression of PRDM5 is related to the clinical pathological parameter lymph node metastasis status, and we speculate that it is related to the WNT signaling pathway and TWIST1. Studies have shown that β-catenin was found in the form of continuous protein methylation after activation and accumulated in the silent PRDM5 cell line. This leads to the activation of the Wnt/β-catenin signaling pathway, downstream LEF/TCF transcriptional activity and the start of EMT process [10]. Many studies have indicated that the WNT signaling pathway can impinge the development of Esophageal squamous cell carcinoma through the EMT pathway [32–35]. Studies have also shown that PRDM5 can bind to the promoter of TWIST1 to affect its transcriptional activity [10]. Studies have shown that patients with esophageal squamous cell carcinoma overexpressing TWIST1 had a poor prognosis, and the increase of TWIST1 can affected the invasion and migration ability of esophageal squamous cell carcinoma [36–38]. Relevant studies have shown that silence of TWIST1 in esophageal squamous cell carcinoma, cervical cancer and non-small cell lung cancer can enhance its drug sensitivity to cisplatin [39–41], and studies have also shown that increased expression of TWIST1 can enhance the radiotherapy resistance of esophageal squamous cell carcinoma cells [42]. We suspect that this may be related to the longer overall survival of patients with high expression of PRDM5 that we have observed after adjuvant treatment after surgery. Patients with high expression of PRDM5 may have lower levels of TWIST1. In future studies, we should further verify the correlation between PRDM5 and TWIST1 at the cellular level. It is best to monitor the blood dynamics of clinical samples of esophageal squamous cell carcinoma to assess their impact on the treatment effect.

Taken together, this work uncovers PRDM5 is a negative prognostic factor in esophageal tumor cell. Future researches should focus on how to conduct clinical detection of methylation sites to predict the prognosis of Esophageal squamous cell carcinoma patients. In the future, more detailed studies are still needed to describe and reveal the underlying mechanism of PRDM5 multiple pathological functions in esophageal squamous cell carcinoma. A comprehensive understanding of the multiple roles of PRDM5 in esophageal squamous cell carcinoma will provide more important clinical value for PRDM5 as a diagnostic indicator, prognostic marker and treatment target for clinical treatment.

## Conclusions

As a tumor suppressor gene in esophageal squamous cell carcinoma, PRDM5 can be used as a biomarker to predict the survival of patients with esophageal squamous cell carcinoma, and the high expression level of PRDM5 is associated with longer OS in patients who underwent postoperative therapy. Furthermore, PRDM5 expression in esophageal squamous cell carcinoma cells may affect WNT/β-catenin signaling pathways, which further affect the esophageal squamous cell carcinoma cell proliferation, migration, and invasion capacity.

## Supplementary Information

**Additional file 1: Supplementary Fig. 1.** A nomogram for predicting the prognosis of patients with esophageal squamous cell carcinoma. T, Tumor size; N, Lymph node metastasis status; TNM, Clinical stag; V, Vascular invasion.

## Data Availability

All the data generated or analyzed during this study are included in this published article. The GSE53622 dataset is available at (https://www.ncbi.nlm.nih.gov/geo/query/acc.cgi?acc=GSE53622) and GSE53624 dataset is available at (https://www.ncbi.nlm.nih.gov/geo/query/acc.cgi?acc=GSE53624) respectively.

## Abbreviations

ESCC: Esophageal squamous cell carcinoma
IARC: The International Agency for Research on Cancer
EMT: Epithelial-Mesenchymal Transition
IHC: Immunohistochemistry
TCGA: The Cancer Genome Atlas
GEO: Gene Expression DataSets
qRT-PCR: quantitative Real-time Polymerase Chain Reaction
MSP: Methylation-specific polymerase chain reaction
TMA: Tissue microarray Confidence intervals
CI: Confidence intervals
HR: Hazard ratio
OS: Overall survival
DFS: Disease-free survival
PBS: Phosphate buffer saline
GAPDH: Glyceraldehyde-3-phosphate dehydrogenase
PRDM5: PR domain zinc finger protein 5
Si-RNA: Small interfering RNA
TCF/LEF: T-cell Factor/Lymphoid Enhancing Factor
BRAF: B-Raf proto-oncogene, serine/threonine kinase
CHIP: Chromatin Immunoprecipitation
HDAC1: Histone deacetylase 1
