# Supplementary figures and images for: Low expression of PRDM5 predicts poor prognosis of esophageal squamous cell carcinoma
Source: BMC Cancer. 2022 Jul 7;22:745. doi: 10.1186/s12885-022-09787-8 (PMC9264607; doi:10.1186/s12885-022-09787-8)

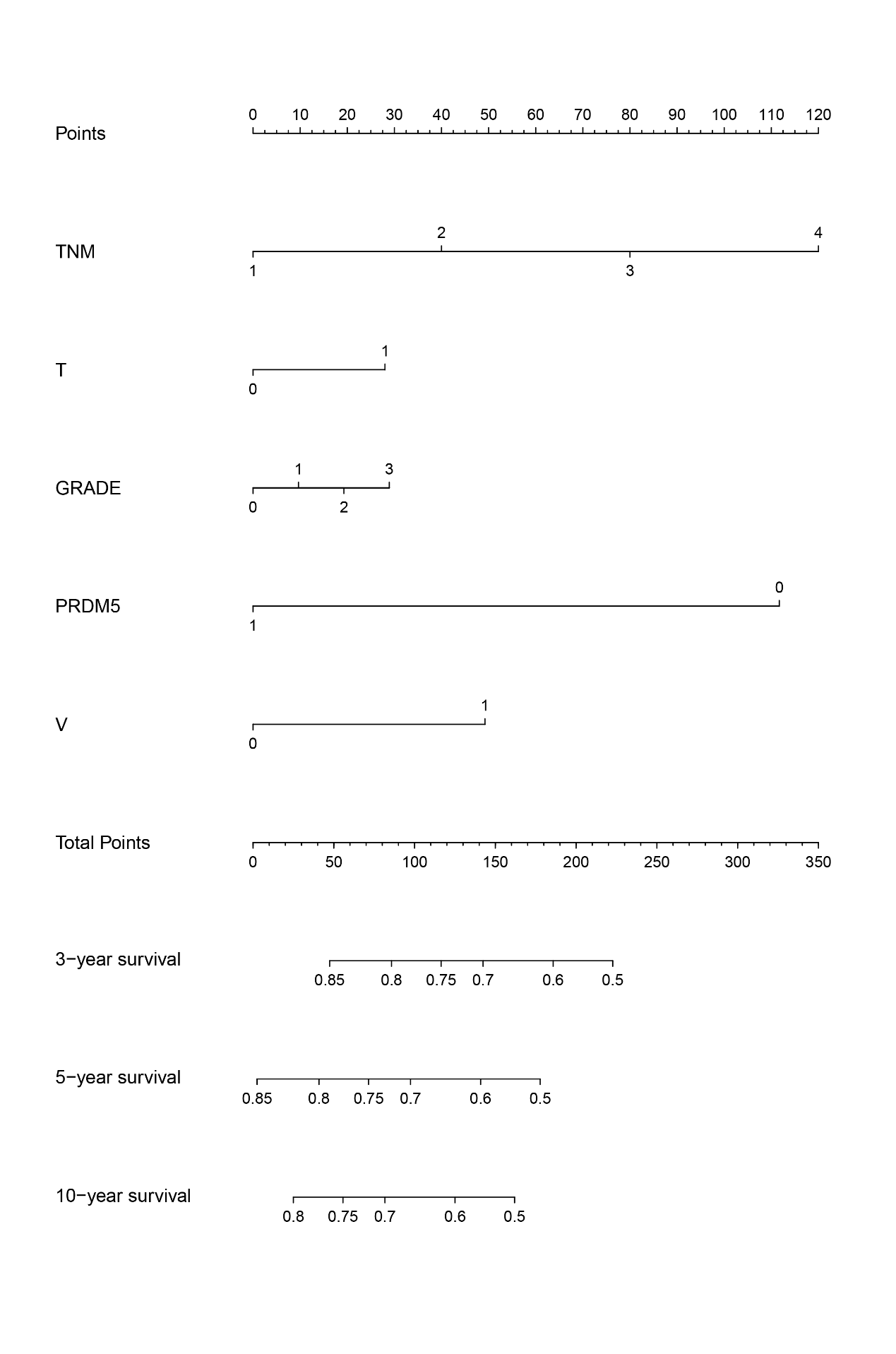

Supplement: Supplementary file 1 — Additional file 1: Supplementary Fig. 1. A nomogram for predicting the prognosis of patients with esophageal squamous cell carcinoma. T, Tumor size; N, Lymph node metastasis status; TNM, Clinical stag; V, Vascular invasion. [file 12885_2022_9787_MOESM1_ESM.jpeg]
